# PARP2 downregulation in T cells ameliorates lipopolysaccharide-induced inflammation of the large intestine

**DOI:** 10.3389/fimmu.2023.1135410

**Published:** 2023-06-30

**Authors:** Máté Bencsics, Bálint Bányai, Haoran Ke, Roland Csépányi-Kömi, Péter Sasvári, Françoise Dantzer, Najat Hanini, Rita Benkő, Eszter M. Horváth

**Affiliations:** ^1^ Department of Physiology, Semmelweis University, Budapest, Hungary; ^2^ UMR7242, Biotechnology and Cell Signaling, CNRS/Université de Strasbourg, Strasbourg, France

**Keywords:** colitis, IBD - inflammatory bowel disease, PARP (poly(ADP-ribose) polymerase, PARP2, T lymphocyte, regulatory (Treg) cell

## Abstract

**Introduction:**

T cell-dependent inflammatory response with the upregulation of helper 17 T cells (Th17) and the downregulation of regulatory T cells (Treg) accompanied by the increased production of tumor necrosis alpha (TNFa) is characteristic of inflammatory bowel diseases (IBD). Modulation of T cell response may alleviate the inflammation thus reduce intestinal damage. Poly(ADP-ribose) polymerase-2 (PARP2) plays role in the development, differentiation and reactivity of T cell subpopulations. Our aim was to investigate the potential beneficial effect of T cell-specific PARP2 downregulation in the lipopolysaccharide (LPS) induced inflammatory response of the cecum and the colon.

**Methods:**

Low-dose LPS was injected intraperitoneally to induce local inflammatory response, characterized by increased TNFa production, in control (CD4Cre; PARP2+/+) and T cell-specific conditional PARP2 knockout (CD4Cre; PARP2f/f) mice. TNFa, IL-1b, IL-17 levels were measured by ELISA, oxidative–nitrative stress was estimated by immunohistochemistry, while PARP1 activity, p38 MAPK and ERK phosphorylation, and NF-kB expression in large intestine tissue samples were examined by Western-blot. Systemic & local T cell subpopulation; Th17 and Treg alterations were also investigated using flowcytometry and immunohistochemistry.

**Results:**

In control animals, LPS induced intestinal inflammation with increased TNFa production, while no significant elevation of TNFa production was observed in T cell-specific PARP2 knockout animals. The absence of LPS-induced elevation in TNFa levels was accompanied by the absence of IL-1b elevation and the suppression of IL-17 production, showing markedly reduced inflammatory response. The increase in oxidative-nitrative stress and PARP1-activation was also absent in these tissues together with altered ERK and NF-kB activation. An increase in the number of the anti-inflammatory Treg cells in the intestinal mucosa was observed in these animals, together with the reduction of Treg count in the peripheral circulation.

**Discussion:**

Our results confirmed that T cell-specific PARP2 downregulation ameliorated LPS-induced colitis. The dampened TNFa production, decreased IL-17 production and the increased intestinal regulatory T cell number after LPS treatment may be also beneficial during inflammatory processes seen in IBD. By reducing oxidative-nitrative stress and PARP1 activation, T cell-specific PARP2 downregulation may also alleviate intestinal tissue damage.

## 1 Introduction

Inflammatory bowel disease (IBD) is a chronic auto-inflammatory disorder; the two major subtypes comprise Crohn’s disease (CD) and ulcerative colitis (UC) (estimated prevalence of 0.3%, respectively) (1, 2). In spite of the intensive research in this area, the etiology of the disease is still not fully elucidated, leading to ineligible therapeutic options available for clinicians and their patients. The rising number of diagnosed IBD cases also underlines the importance of a greater understanding of this disease and the introduction of more efficient therapeutic alternatives (3, 4).

In IBD patients, a chronic pathological inflammation is present in the intestinal mucosa with several acute exacerbations leading to mucosal damage and increased intestinal permeability (5–7). The immune reaction can be evoked by the normal intestinal microflora. Increased oxidative-nitrative stress is another hallmark of intestinal inflammation that together with inflammatory signal transduction induces further cellular damage and the activation of poly(ADP-ribose) polymerase 1 and 2 (PARP1 and PARP2) (8, 9).

T cell-specific immune response has inevitable role in the pathogenesis of IBD. Activated suppressor and helper T cells in the mucosa and the lamina propria produce pro-inflammatory cytokines and maintain inflammation. The increased production of TNFα is a common characteristic in IBD patients making it a target for immunological therapies. IL-17 together with TNFα plays important role in the pathological regulation of inflammatory gene expression. In this inflammatory milieu, upregulation of helper T cells 17 (Th17) and downregulation of regulatory T cells (Treg) can be observed, which can further contribute to the maintenance and escalation of inflammation. Modulation of T cell function is a new and emerging field in the IBD research and therapy.

Based on recent studies, PARP2, member of the adenosine diphosphate ribosyl transferase (ADPRT) enzyme superfamily exerts a decisive effect on immune reactions and plays an important role in T cell homeostasis (10). PARP2 is activated upon DNA damage, similar to the more characterized member of the ADPRT enzyme superfamily, PARP1. Both enzyme synthesizes poly(ADP-ribose) (PAR) polymers from nicotinamide adenine dinucleotide (NAD^+^) and ligates them to mainly nuclear proteins. Although PARP1 and PARP2 heterodimerize and share several common nuclear binding partners, their characteristics and functions are similar but not fully overlapping. While PARP1 targets DNA nicks, PARP2 favors DNA gaps. PARP1 preferentially modifies histone H1, while for PARP2 mainly histone H2B is the major acceptor (8, 11, 12). In comparison to PARP1, the highest catalytic activity member of ADPRT enzyme superfamily, whose role was widely investigated in Crohn’s disease, PARP2 was rarely investigated.

Recent findings suggest that PARP2 plays important role in T cell homeostasis. PARP2 also plays role in the development of T cells, described in global PARP2 knockout mice. These mice had reduced number of thymocytes in the thymus due to the decreased survival of double positive T cells, showing no significant decrease in double negative T cell maturation or cell proliferation (10). In an experimental autoimmune encephalomyelitis model, mice lacking PARP2 had decreased Th1 and Th17 infiltration of the central nervous system, showing its role in the migration and activation of T cell subpopulations (13). Molecular targets regulated by PARP2 influencing T cell homeostasis are less characterized. In a myeloid/osteoclast specific conditional PARP2 knockout mouse model, reduced production of various chemokines, such as CCL3, was observed in a breast cancer metastasis model, which was accompanied by increased Treg and reduced Th1 numbers (14). Similar to the global PARP2 knockout mice, the total number of T cells in the spleen was reduced in T cell‐specific conditional PARP2 knockout mice, due to the reduced number of double positive T cells. On the other hand, no change in more matured single positive (CD4 or CD8) T cell number was reported. The T cell response for T dependent antigen of these mice was not altered, the immune reaction induced by vaccinia virus infection with or without prior vaccination showed minimal changes (15).

Our aim was to investigate the potential benefit of T cell-specific PARP2 deficiency in the lipopolysaccharide (LPS) -induced inflammatory response characterized by increased TNFα in large intestine.

## 2 Methods

### 2.1 Animals

On the basis of the T cell-specific PARP2 knockout mice provided courtesy of Professor Dantzer and the University of Strasbourg, our group established breeding strains of control (CD4Cre; PARP2^+/+^) and T cell-specific conditional PARP2 knockout (T-cell-PARP2-KO) (CD4Cre; PARP2f/f) mice. The present investigation conformed to the EU Directive 2010/63/EU and to the Guide for the Care and Use of Laboratory Animals published by the US National Institutes of Health (NIH Publication No. 85-23, revised 1996). The study was reviewed and approved by the Scientific Ethical Committee on Animal Experimentation (Hungary) and by the Institutional Ethics Committee of Semmelweis University (Reference No. PE/EA/1652-7/2018). Animals were kept under standard conditions with 12-hour-long light and dark cycles and were allowed access to standard laboratory rat chow and water ad libitum during the experimental period.

### 2.2 Induction of intestinal inflammation

The experiments were performed on male mice aged 14 to 18 weeks. Control (CD4Cre; PARP2+/+) and T cell-specific PARP2 knockout (T-cell-PARP2-KO) animals were further divided into two groups, one received intraperitoneal LPS injection (2 mg/kg dose of E. coli LPS, 1 mg/ml concentration) and the other remained untreated. Each group (control, control + LPS, T-cell-PARP2-KO, T-cell-PARP2-KO + LPS) consisted of 12 animals. These treatments were followed by a 6-hour-long observational period (continuous presence of a researcher, condition assessment every 30 minutes). Under deep anesthesia (2,2,2-tribromo-ethanol, 0.375 mg/g intraperitoneal) the blood of the animals was collected through the cannulation of the inferior vena cava. The circulatory system of the animals was perfused with saline solution. Subsequently, samples were collected from the cecum and colon of the animals. These tissues were placed in phosphate-buffered formaldehyde or snap frozen in fluid nitrogen and stored at -80°C.

### 2.3 Determination of T cell subpopulations

The relative number of T cell subpopulations in the heparinized blood samples was determined by four-channel flow cytometry. Antibodies were obtained from Thermo Fisher Scientific (Waltham, MA, USA) and were used to target CD3 (ANTI-MO CD3 17A2 FITC 100UG; 11-0032-82; RRID: AB_2572431), CD4 (ANTI-MO CD4 RM4-5 PERCP-CYN5.5 100UG; 45-0042-82; RRID: AB_1107001), CD25 (ANTI-MO CD25 PC61.5 PE 100UG; 12-0251-82; RRID: AB_465607) and CD196 (ANTI-MO CD196 SIRX6 EF660 100UG; 50-7196-82; RRID: AB_11219682) antigens. After the identification of the CD3 and CD4 positive T cell population the ratio of Th17/Th (CD3+ CD4+ CD196+/CD3+ CD4+) and Treg/Th (CD3+ CD4+ CD25+/CD3+ CD4+) were determined by marking their specific cell membrane markers. Missing values are due to low total cell counts.

### 2.4 Markers of local intestinal inflammation

#### 2.4.1 Inflammatory cytokines, inflammation

Deep-frozen intestinal tissue samples from the colon of the animals were homogenized in RIPA buffer (Bio-Rad Laboratories, Hercules, CA, USA) containing protease-phosphatase inhibitor cocktail (Roche, Basel, Switzerland) using a tissue homogenizer with constant cooling. The protein concentrations of the obtained homogenate supernatants were quantified using the BCA assay (Thermo Fisher Scientific). Inflammatory cytokine concentrations from cecum and colon homogenates were measured using the ELISA method (IL17 - Invitrogen Mouse IL-17 ELISA Kit BMS6001, TNFα - Invitrogen Mouse TNFα ELISA Kit BMS607-3 and IL1β - Invitrogen Mouse IL-1 beta ELISA Kit BMS6002) (Thermo Fisher Scientific), and normalized to total protein content. N=10-12/group. Missing values are due to low protein concentration. The level of mucosal inflammation was examined in hematoxylin-eosin stained histological sections according to an inflammatory score system (**Supplementary Table 2**) (16). For immunohistochemistry intestinal tissue samples from the cecum and the colon of the animals were fixed in 4% neutral buffered paraformaldehyde solution, embedded in paraffin, and 5 µm thick histological sections were sliced. Following deparaffinization, epithelial localization of TNFα was analyzed by immunohistochemistry (pH6 citrate buffer antigen retrieval for 17 min, TNFα antibody, SAB4502982, Sigma-Aldrich, St. Louis, MO, USA; RRID : AB_10746474 Secondary labeling was achieved by HRP-linked anti-rabbit polyclonal horse antibodies (MP-7401-15, Vector Laboratories, California, USA), which was visualized by brown-colored diamino-benzidine (DAB, SK-4100, Vector Laboratories, California, USA). Blue-colored hematoxylin (H-3404, Vector Laboratories, California, USA) was utilized as counterstaining. Images of the immunolabeled intestinal tissue sections were captured by Nikon Eclipse Ni Microscope (Nikon Instruments, Amstelveen, The Netherlands) with a 20× objective lens, using a Nikon DS-RI2 camera (Nikon Instruments) and NIS-Elements BR imaging software (Nikon Instruments).

#### 2.4.2 Intracellular inflammatory signal transduction

Members of the intracellular inflammatory signal transduction were examined by Western blotting in four to five animals per group. Supernatants of the lysates were boiled at 100°C for 5 minutes in reducing SDS sample buffer and equal protein mass of each sample was run on 4-15% (w/v) gradient polyacrylamide gels (Bio-Rad, Hercules, California, USA) and separated proteins were transferred to nitrocellulose membrane (Bio-Rad, Hercules, California, USA). After blocking for 10 minutes in EveryBlot blocking buffer (Bio-Rad, Hercules, California, USA), membranes were incubated with the following monoclonal antibodies: p38 MAPK (#9212S; RRID: AB_330713), phospho-p38 MAPK (#4511S; RRID: AB_2139682), p44/42 ERK (#4695S; RRID: AB_390779), phospho-p44/42 ERK (#4370S; RRID: AB_2315112), NF-κB p65 (#8242S; RRID: AB_10859369),(Cell Signaling, Danvers, MA, USA) in 1:1000 dilution overnight at 4°C. Bound antibody was detected with enhanced chemiluminescence using horseradish peroxidase-conjugated anti–rabbit-IgG (from donkey) secondary antibody (GE Healthcare, Chicago, Illinois, USA, NA934V) in 1:5000 dilution (1 hour, room temperature). Alpha-smooth muscle cell actin was detected with the use of anti-actin antibody (ab7817, Abcam, Cambridge, United Kingdom; RRID: AB_262054). Band intensity was quantified by the ImageJ software (ver. 1.53o). The films were scanned at 600 dots per inch in TIF format. Each band was individually selected and the peak area was acquired and quantified as arbitrary area values of each histogram thrice. For data normalization, we used actin as a housekeeping protein. Data of multiple membranes were analyzed following normalization.

#### 2.4.3 Oxidative-nitrative stress and PARP activation in intestinal tissues

Tissue PARP activity (level of protein PARylation) was examined by Western blotting in six to eight animals per group. Supernatants of the lysates were boiled at 95°C for 5 minutes in reducing SDS sample buffer and equal protein mass of each sample was run on 4–12% (w/v) gradient polyacrylamide gels and separated proteins were transferred to nitrocellulose membrane (Invitrogen). Aspecific labeling of the membranes was prevented by incubation at room temperature with 10% non-fatty dry milk solution (1 h). Membranes were then incubated overnight (at 4°C) with the anti-PAR antibody 1:1000 in 1% milk, (ab14459, Abcam, Cambridge, UK; RRID: AB_301239). Following the washing procedure, membranes were incubated for 1h (at room temperature) with horseradish peroxidase-conjugated secondary antibody (1:1000 in 1% milk) (goat anti-mouse HRP Invitrogen catalog #31460). Alpha-smooth muscle actin was used as a loading control (ab7817 1:10000 in 1% milk; Abcam, Cambridge, United Kingdom; RRID: AB_262054). Super Signal West Pico Plus (Thermo Fisher Scientific) chemiluminescent substrate was used to develop the immunoreactive protein bands. The Bio-Rad Image Lab Software (Bio-Rad Laboratories) was used to analyze the intensity of the developed bands, measuring adjusted integral density, background was the same for every band. Within each sample, the intensity of the bands of the primary targets was normalized to that of the alpha-smooth muscle cell actin on the same blot. Data of multiple membranes were analyzed following normalization.

Epithelial localization of protein PARylation was analyzed by immunohistochemical staining of the previously formalin-fixed and paraffin-embedded colon segments (0,15% PBS proteinase K antigen retrieval for 12 min, anti-PAR mouse monoclonal antibody (ab14459, 1:500, Abcam; RRID: AB_301239). Epithelial nitrative stress was also estimated by the immunohistochemical labeling of 3-nitrotyrosine (3-NT) nitrosamine stress marker (0,15% PBS proteinase K antigen retrieval for 12 min, 3-nitrotyrosine antibody 06-284 rabbit polyclonal 1:250, Merck, Massachusetts, USA; RRID: AB_310089) in 4-5 mice/group. The intestinal localization of regulatory T cells was examined by the immunohistochemical labeling of FoxP3 (pH3 citrate buffer antigen retrieval for 17 min, FoxP3 (14-5773-82) rat monoclonal IgG2a kappa antibody 1:500, Invitrogen, Massachusetts, USA; RRID: AB_467576 and isotype control (14-4321-82) rat IgG2a kappa antibody 1:500, Invitrogen, Massachusetts, USA; RRID: AB_470105). During immunohistochemistry, secondary labeling was achieved by HRP-linked anti-rabbit polyclonal horse antibodies (MP-7401-15, Vector Laboratories, California, USA), which was visualized by brown-colored diamino-benzidine (DAB, SK-4100, Vector Laboratories, California, USA). Blue-colored hematoxylin (H-3404, Vector Laboratories, California, USA) was utilized as counterstaining. Images of the immunolabeled intestinal tissue sections were captured by Nikon Eclipse Ni Microscope (Nikon Instruments, Amstelveen, The Netherlands) with a 20× objective lens, using a Nikon DS-RI2 camera (Nikon Instruments) and NIS-Elements BR imaging software (Nikon Instruments). In the case of NT labeling, staining intensity was estimated by measuring non-calibrated optical density of the brown color (DAB) in the mucosa. In the case of FoxP3, positively stained cells were counted in the whole cross-section of the intestinal segment, and the number was normalized to the epithelial area using ImageJ Software (National Institutes of Health, Bethesda, MA, USA).

### 2.5 Statistical analysis

Statistical analysis was performed by GraphPad statistical software package (GraphPad Software, La Jolla, CA). Statistical probes were based on two-way ANOVA with Tukey’s *post hoc* test, and variables with non-Gaussian distribution (Shapiro-Wilk test) were log-transformed for analysis. p<0.05 was considered statistically significant. Data are presented as mean±-standard deviation (SD) in the case of normal distribution variables, and median [IQR] in the case of non-Gaussian distribution. N represents the number of animals per group from 3-5 independent experiments. Raw data of each analysis can be found in **Supplementary Table 1**.

## 3 Results

### 3.1 The local inflammatory response in the colon and in the cecum

#### 3.1.1 Inflammatory cytokines, oxidative-nitrative stress and inflammation

**Figure 1:**
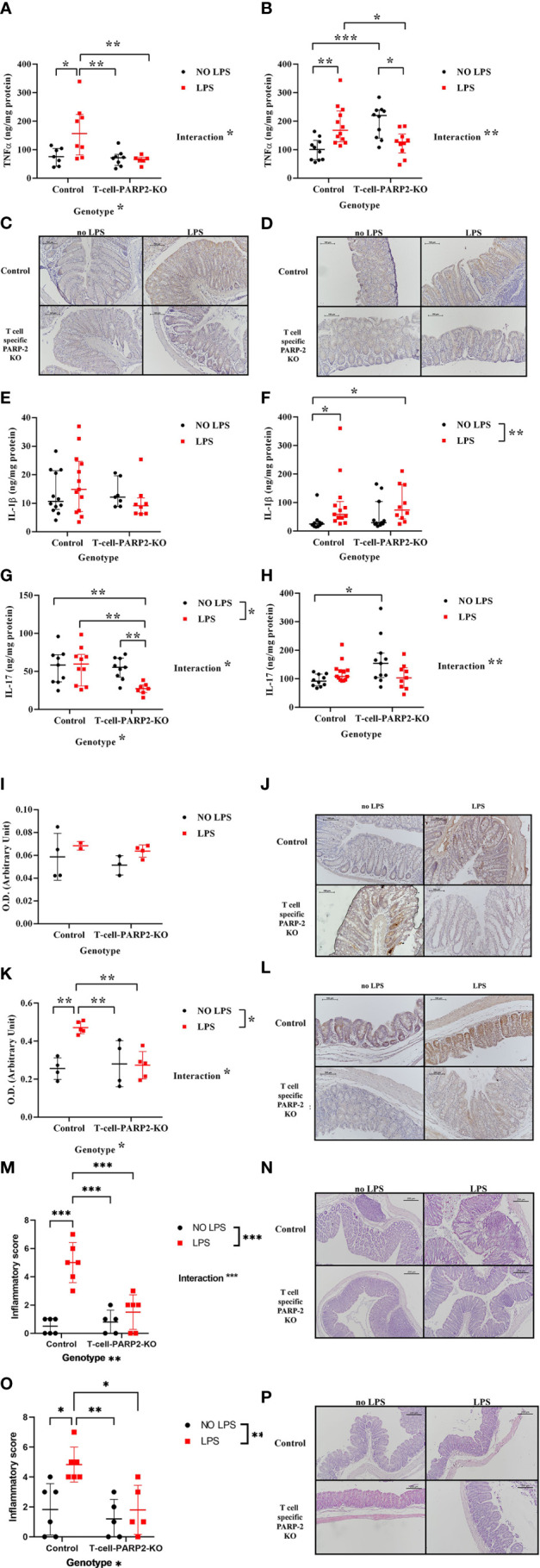
Tissue inflammatory response of control and T-cell-PARP2-KO mice with and without LPS treatment. **(A)** Tissue concentration of TNFα in the colon. LPS treatment increased TNFα level only in the control group. **(B)** Tissue concentration of TNFα in the cecum. LPS administration caused genotype-specific alteration of TNFα concentration; while it caused elevation in the control group, it led to a decrease in the T-cell-PARP2-KO group. **(C, D)** Representative images of colon and cecum tissues labeled with anti-TNFα antibody. Brown colored diaminobenzidine represents specific staining, whereas blue colored hematoxylin serves as counterstaining. **(E)** Tissue concentration of IL-1ß in the colon. No significant changes were observed. **(F)** Tissue concentration of IL-1ß in the cecum. Similar to TNFα, an increment in IL-1ß level after LPS administration was detected only in the control group. **(G)** Tissue concentration of IL-17 in the colon. While in the control group no change in IL-17 concentration was detected in response to LPS, in T-cell-PARP2-KO mice a marked reduction was observed. **(H)** Tissue concentration of IL-17 in the cecum. T-cell-PARP2-KO mice showed a higher level of IL-17. Lines represent median[IQR]. Two-way ANOVA (genotype and LPS treatment) with Tukey’s *post-hoc* test was performed on logarithm-transformed data. *: p<0.05, **: p<0.01, ***: p<0.001; N=10-12/group. **(I)** Evaluation of immunohistochemical labeling of 3-nitrotyrosine in the colon. No significant increase was observed in any groups following LPS treatment. **(J)** Representative images of colon labeled with anti-nitrotyrosine antibody. Brown colored diaminobenzidine represents specific staining, whereas blue colored hematoxylin serves as counterstaining. The scale bar is 100 µm. **(K)** Evaluation of immunohistochemical labeling of 3-nitrotyrosine in the cecum. LPS administration induced increased nitrative stress only in the control group. **(L)** Representative images of cecum labeled with anti-nitrotyrosine antibody. **(M, O).** Histological inflammatory score of the colon and the cecum. Two-way ANOVA showed increased inflammation after LPS treatment, which was only significant in the control group. **(N, P).** Representative histological sections of colon and cecum stained with hematoxylin-eosin staining. Lines represent mean ± SD Two-way ANOVA (genotype and LPS treatment) with Tukey’s *post-hoc* test, *: p<0.05,**: p<0.01,***: p<0.001, N=2-6 animals/group.

As increased TNFα production is a hallmark of IBD and a common characteristic with LPS induced inflammatory reaction, first we examined the potential effect of T cell-specific PARP2 downregulation on the intestinal tissue concentration of this cytokine with or without LPS treatment. In the colon, the tissue level of TNFα was similar in the control and in the T-cell-PARP2-KO groups when measured without LPS treatment. While LPS treatment induced an elevation in TNFα concentration in control animals as expected, no induction of TNFα was detected in T-cell-PARP2-KO animals (**Figure 1A**). In the cecum, LPS treatment also resulted in a marked increase of TNFα concentration in the control mice. In contrast, TNFα level was reduced after LPS administration in T-cell-PARP2-KO animals, these mice having initially a higher basic level of TNFα compared to the control animals (**Figure 1B**). The localization of TNFα in the intestinal wall was examined by immunohistochemistry, showing positive staining of the mucosa and immune cells (**Figures 1C, D**). The other characteristic pro-inflammatory cytokine, IL-1ß, despite having similar trends, showed no difference related to genotype or LPS treatment in the colon (**Figure 1E**). In the cecum, LPS treatment induced elevated IL-1ß concentration in the control group but not significantly in the T-cell-PARP2-KO animals (**Figure 1F**). IL-17, which is a key regulator of Th17-dominated immune response, was not altered by LPS treatment in the large intestine of control mice (**Figures 1G, H**). In the colonic tissues of T-cell-PARP2-KO animals, IL-17 concentration showed depression after LPS administration (**Figure 1G**). Similar to TNFα, IL-17 levels were also higher in untreated T-cell-PARP2-KO mice compared to control mice (**Figure 1H**). Increased oxidative-nitrative stress is also a hallmark of inflammatory processes. We estimated local epithelial nitrative stress by the immunohistochemical labeling of 3-nitrotyrosine (NT). In the colon, LPS treatment failed to induce a significant elevation of nitrative stress in both groups (**Figures 1I, J**). NT staining intensity was significantly increased in the cecum of control animals following LPS injection, which was abolished by the downregulation of PARP2 in T-cells (**Figures 1K, L**). LPS induced tissue inflammation was also supported by the evaluation of hematoxylin-eosin stained tissue sections both in the colon (**Figures 1M, N**) and in the cecum (**Figures 1O, P**) of the control animals, whereas no signs of significant inflammation was observed in the T-cell-PARP2-KO animals.

#### 3.1.2 Inflammatory signal transduction in the colon

**Figure 2:**
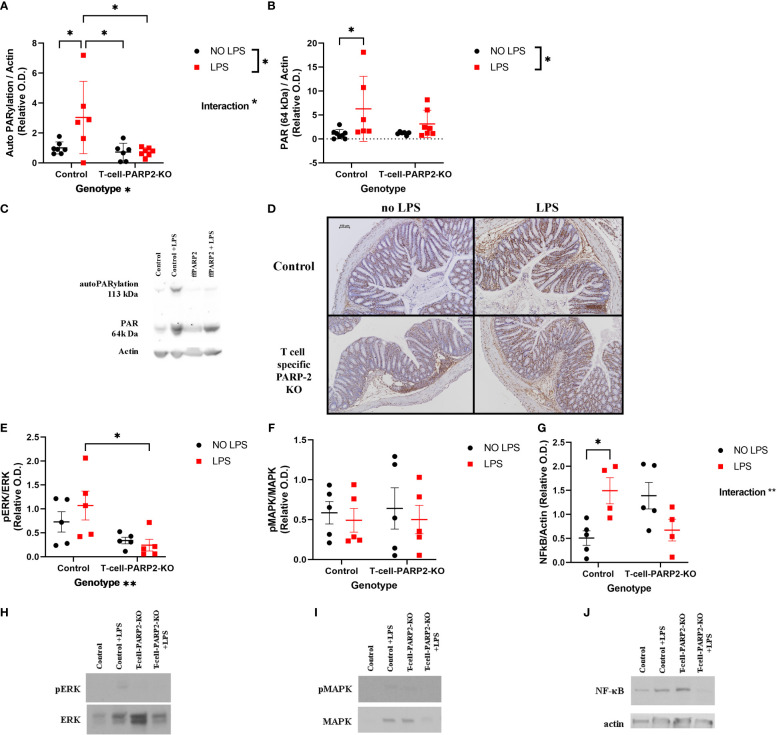
Inflammatory signal transduction in the colon. **(A)** PARP1 activation. PARylation at 113-120 kD; densitometric analysis of anti-PAR Western blots was carried out and normalized integrated OD was plotted. The degree of PARP1 auto-PARylation was the highest in the LPS-treated control animals. **(B)** General protein PARylation. PARylation at 64 kDa. No significant difference was observed. **(C)** Representative Western-blot images of membranes stained with anti-PAR antibody. **(D)** Representative colon tissue sections stained with anti-PAR antibody. Brown-colored diaminobenzidine represents specific staining, the counterstaining is blue-colored hematoxylin. The scale bar is 100 µm. **(E)** ERK phosphorylation. T-cell-PARP2-KO mice had a lower level of ERK phosphorylation after LPS treatment compared to controls. **(F)** p38 MAPK phosphorylation. No significant difference was observed. **(G)** NF-κB expression. Two-way ANOVA proved the interaction between genotype and LPS treatment. **(H)** Representative Western-blot images stained with phospho-ERK and ERK antibodies. **(I)** Representative Western-blot images stained with phospho-MAPK and MAPK antibodies. **(J)** Representative Western-blot images stained with NF-κB and actin antibodies. Lines represent mean ± SD, two-way ANOVA (genotype and LPS treatment) with Tukey’s *post-hoc* test, *: p<0.05, N=4-8 animals/group.

Signal transduction mechanisms that may diversify the course of reaction of control and T-cell-PARP2-KO animals to LPS treatment were examined in the colon. PARP1 activation was examined by Western Blot analysis, measuring the level of total protein PARylation on membranes stained with an anti-PAR antibody. Protein lines at 113-120 kDa correspond to auto-PARylation of the enzyme reflecting enzyme activity and the line at 64 kDa, with the strongest labeling reflecting general protein PARylation was examined. A significant increment in the control group following LPS treatment was observed in the autoPARylation level of PARP1, whereas PARP1 activity showed no remarkable alteration in the T-cell-PARP2-KO mice (**Figures 2A, C**). General protein PARylation was also elevated in control animals after LPS treatment (**Figures 2B, C**). Immunohistochemical labeling of PAR showed a nuclear localization with a similar trend to protein PARylation at 64 kDa on the Western blot (**Figure 2D**). Activation of ERK and p38 MAPK, the two other important regulators of NF-κB expression were investigated by measuring their level of phosphorylation. After LPS treatment T-cell-PARP2-KO animals had a significantly lower level of ERK phosphorylation compared to control mice (**Figures 2E, H**). No significant difference was observed in the case of MAPK phosphorylation (**Figures 2F, I**). Similarly, we saw no difference between experimental groups in the case of NF-κB expression; however, two-way ANOVA analysis revealed a significant interaction between genotype and LPS treatment, suggesting that control and T-cell-PARP2-KO animals had a different reaction to LPS administration; while in the control group we saw the increasing tendency for NF-κB expression, in the T-cell-PARP2-KO group we observed decreasing tendency (**Figures 2G, J**).

### 3.2 Systemic T cell response

**Figure 3:**
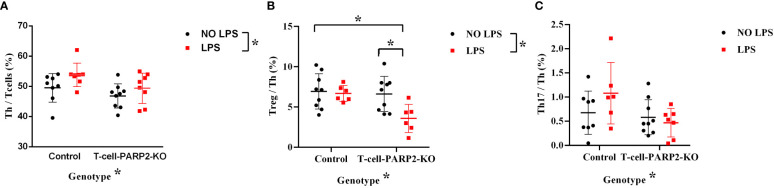
The ratio of T cell subpopulations in the peripheral blood in control and T-Cell-PARP2-KO mice with and without LPS treatment. **(A)** The ratio of helper T cells compared to total T cell number (Th/T). The T-cell-PARP2-KO group had a lower Th/T ratio and LPS treatment induced an increased Th/T ratio independent of genotype. **(B)** Ratio of regulatory T cells compared to helper T cells (Treg/Th). LPS treatment resulted in reduced Treg/Th ratio only in the T-cell-PARP2-KO group. **(C)** Ratio of Th17 cells compared to helper T cells (Th17/Th). No significant difference was detected due to low Th17 cell numbers. Lines represent mean ± SD, Two-way ANOVA with Tukey’s *post-hoc* test *: p<0.05; N=6-9 animals/group.

The changes in peripheral blood T cell subpopulations were investigated to characterize systemic T cell response. The ratio of helper T cells to total T cell number (Th/T: CD3, CD4/CD3) measured by flow-cytometry was lower in T-cell-PARP2-KO mice compared to controls. LPS treatment induced an increment of the Th/T ratio independent of the genotype (**Figure 3A**). The ratio of Treg/Th-cells (CD3,CD4,CD25/CD3,CD4) was similar in both genotypes without LPS treatment. While LPS provoked no change in control mice (6.9 ± 2.2; 6.7 ± 1%), a significant decrease was observed in the T-cell-PARP2-KO group (6.6 ± 2.2 vs. 3.6 ± 1.8%, p< 0.05) (**Figure 3B**). The ratio of Th17/Th (CD3,CD4,CD196/CD3,CD4) was below 1% thus did not show any detectable difference (**Figure 3C**).

### 3.3 Regulatory T cells in the large intestine

**Figure 4:**
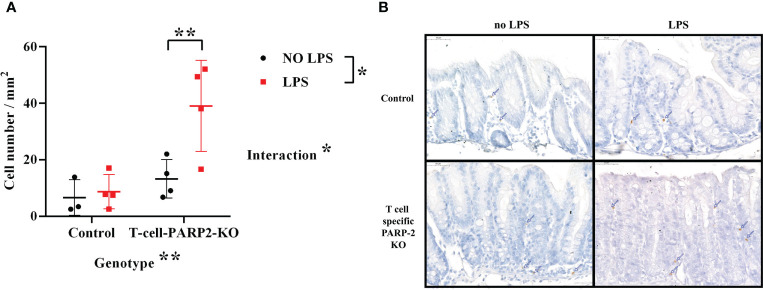
Regulatory T cells in the large intestinal mucosa. **(A)** Number of FoxP3 positive cells in the cecum per mm^2^. The number of regulatory T cells was increased after LPS treatment only in the T-cell-PARP2-KO group. **(B)** Representative images of FoxP3 labeled colonic sections. Brown-colored diaminobenzidine represents specific staining, the counterstaining is blue-colored hematoxylin. Blue arrows show positively stained cells. The scale bar is 50µm. Lines represent mean ± SD, Two-way ANOVA, *: p<0.05, **p<0.001; N=3-4 animals/group.

The reduction of Treg cell number in the peripheral circulation was accompanied by dampened intestinal inflammatory reaction in T-cell-PARP2-KO mice after LPS treatment. Such a fact may suggest that the inflammatory response following LPS treatment induces the recruitment of these cells into intestinal tissues in T-cell-PARP2-KO mice. In the cecum, the number of FoxP3 positive cells increased notably in T cell-specific PARP2 KO animals following LPS treatment, while it remained unchanged in control mice. No significant change was observed in the colon (data not shown) (**Figure 4**).

## 4 Discussion

Previous studies showed elevated PARP1 activity in the inflamed colon of the chemically and genetically induced colitis animal models with an increased level of mucosal protein PARylation or ex vivo enzyme activity. The beneficial effect of PARP1 inhibition was also confirmed in various animal models including dinitrobenzene sulfonic acid (DNBS) and trinitrobenzene sulfonic acid (TNBS) induced acute and chronic colitis and spontaneous chronic colitis developed in IL-10 knockout mice (17–20). On the other hand, the few available human studies suggested a more complex role of PARP1 in the pathogenesis of the disease. Markowitz et al. in 1988 showed that isolated mononuclear cells of IBD patients exhibited reduced hydrogen peroxide-induced PARP1 activation and even the involvement of their first-degree relatives was raised. They suggested that impaired DNA repair may play a role in the pathogenesis of the disease (21). A few years later, autoantibodies against PARP1 (zinc-finger motifs F1 and F2) were identified in Crohn’s disease patients and the level of the specific autoantibody showed a correlation with the Crohn’s disease activity index (CDAI) (5, 22). In our previous study examining the PARP1 activation of colonic tissues in pediatric patients, we observed an elevated mRNA expression of PARP1, however, reduced PARP1 protein level and activity (23).

Less is known about the role of PARP2 in IBD. One available study showed that inhibition of PARP2 expression by antisense nucleotide was also found effective in ameliorating colitis in IL-10 deficient mice. Although both PARP1 and 2 are involved in the development and regulation of T cells, PARP2 has a more crucial role in T cell homeostasis. Downregulation of PARP2 may change T cell response to acute and chronic inflammatory stimuli (15, 24). Th1 and Th17 infiltration, key components of the pathogenesis in IBD and other autoimmune diseases, are suppressed by the downregulation of PARP2 (13). T cell-specific PARP2 knock-out mice also show altered immune reaction (15).

In the present study, T cell-specific PARP2 downregulation was sufficient to weaken the intestinal inflammatory reaction induced by intraperitoneal low dose LPS. Control animals with intact PARP2 function exhibited increased TNFα and IL-1β as expected. Meanwhile, inflammatory cytokine levels including TNFα, IL-1β, and IL-17 were either markedly reduced or not altered in response to LPS induction in T-cell-PARP2-KO animals. Effector T cells produce several inflammatory cytokines playing substantial roles in the pathogenesis of IBD. TNFα as one of the most well-established key mediators of immune reactions is frequently targeted in specific immune therapies for IBD, e.g. by infliximab. IL-17 is an important cytokine affecting the activation of Th17 cells and therefore contributes to two very important features of Crohn’s disease: chronic inflammation and granuloma formation (6, 7).

Our results proved the reduced intestinal inflammatory reaction after LPS treatment in the T-cell-PARP2-KO mice, which was confirmed by the evaluation of the cellular inflammatory cascade activation. Oxidative-nitrative stress of the mucosa estimated by the immunohistochemical labeling of NT was neither increased in these animals after LPS. PARP1 activation also followed this pattern; LPS administration was able to induce PARP1 activation in control mice, but failed to do so in T-cell-PARP2-KO animals, most probably due to the dampened oxidative stress and TNFα production. Besides PARP1 activation MAPK pathways are also involved in the regulation of the pro-inflammatory gene transcription factor, NF-κB expression (25, 26). In our model, T-cell-PARP2-KO mice exhibited lower level of LPS-induced ERK phosphorylation, compared to controls. All these differences between the two genotypes led to a genotype-dependent NF-κB expression change after LPS treatment.

In general, LPS administration induces T cell activation in two major ways. LPS is recognized by Toll-like receptors (TLR) either on antigen presenting cells leading to classic T cell activation or on naïve T cells inducing bystander T cell activation, which is T cell receptor-independent. Both pathways result in cytokine production and Th cell response. We found an increased Th/T cell ratio 6 hours after LPS treatment, independent of genotype, suggesting the presence of Th activation in all animals. Intraperitoneal LPS also causes intestinal inflammation in a similar pathway. Resident tissue macrophages recognize LPS and bind to Toll-like receptor 4 (TRL4), resulting in TNF production. Intestinal epithelial cells respond to the TNF signal with NFκB signaling activating caspase-dependent apoptosis. More recent publications suggest a pathway, where LPS binds to TRL4 of the epithelial cells directly, resulting in PGE_2_ secretion of the epithelial cells, and subsequent leukocyte activation (27, 28).

PARP2 has a decisive effect on T cell maturation both in the thymus and in the periphery. In the thymus, PARP2 is important for the survival of double-positive thymocytes, thus PARP2 knockdown leads to reduced T cell number (29). The lower Th/T ratio in T-cell-PARP2-KO mice might be involved in the weakened intestinal cytokine production of these animals after LPS treatment. On the other hand, T-cell-PARP2-KO mice had a similar cytokine production pattern without LPS treatment, despite their lower Th/T ratio.

Th17 cells themselves are also capable of the direct recognition of LPS that induces among other cytokines IL-17 production (30). Due to the low number of Th17 cells in the peripheral circulation, its alteration could not be detected in the present study. Nevertheless, T-cell-PARP2-KO mice had lower IL-17 production after LPS treatment, suggesting the reduced local activation of Th17 cells. It can be the result of their impaired activation, or the suppression by regulatory T cells. Tregs can also selectively express TLR4, thus be activated by LPS directly, when there is no TCR engagement (30). No significant intestinal alteration of Treg number was observed in control animals 6 hours after LPS treatment; however, an increase was found in the mucosa of T-cell-PARP2-KO mice. This can be the additive result of enhanced local Treg expansion in response to LPS stimulus and the recruitment of extra-intestinal Tregs. In parallel, we found a lower number of Tregs in the peripheral circulation of these animals after LPS treatment, suggesting the presence of increased recruitment.

In conclusion, the intraperitoneal injection of LPS was capable of inducing acute colitis in our experimental animals. The reduced elevation of TNFα production and the decreased tissue concentration of IL-17 found in T cell-specific PARP2 knockout mice may suggest the protective effect of PARP2 suppression in the intestinal inflammatory response. PARP2 downregulation in T cells also led to reduced oxidative-nitrative stress and PARP1 activation that was accompanied by altered ERK and NF-κB activation. The differences in peripheral T cell response and intestinal Treg migration may also participate in the reduced inflammatory response in these animals. Our results suggest that PARP2 suppression in T cells is protective in LPS-induced colitis. As the cellular and humoral alterations in LPS-induced colitis are similar to the changes seen in IBD, especially during the initiation of the disease (27) and in its acute exacerbations, T cell-specific PARP2 inhibition might be beneficial in these conditions. Several PARP inhibitors with different selectivity inhibit both PARP1 and PARP2 enzymes are used in the treatment of various solid tumors, primarily with BCRA mutations. The possible use of these PARP inhibitors for other indications, including inflammatory diseases and IBD has been also suggested (31). On the other hand, the side effects and the possible mutagenic effect of generic PARP inhibition casted doubts on their applicability outside of the field of oncology for decades. According to our results, the suppression of PARP2 enzyme only in T cells may also possess therapeutic potential in intestinal inflammation. With the recent results of the exponentially growing research field of targeted drug delivery systems (32), the T cell-specific inhibition of PARP might be available alternative in the near future.

## Data availability statement

The original contributions presented in the study are included in the article/**Supplementary Material**. Further inquiries can be directed to the corresponding author.

## Ethics statement

The animal study was reviewed and approved by Scientific Ethical Committee on Animal Experimentation (Hungary) and by the Institutional Ethics Committee of Semmelweis University (Reference No. PE/EA/1652-7/2018).

## Author contributions

The authors contributed to this study and recent manuscript as follows: MB: literature search, experiments, data collection, data analysis, figures, manuscript writing. BB: literature search, experiments, data collection, data analysis, figures, images, reviewing manuscript. HK: literature search, experiments, data collection, data analysis, manuscript writing. RC-K: data collection, data analysis, reviewing manuscript, funding. PS: data collection, data analysis, reviewing manuscript. FD: animal model, conceptualization, reviewing manuscript. NH: animal model, conceptualization, reviewing manuscript. RB: literature search, experiments, data collection, reviewing manuscript. EH: Study design, literature search, conceptualization, experiments, data collection, data analysis, manuscript writing, funding. All authors contributed to the article and approved the submitted version.
